# miR-1 Inhibits the Ferroptosis of Chondrocyte by Targeting CX43 and Alleviates Osteoarthritis Progression

**DOI:** 10.1155/2023/2061071

**Published:** 2023-06-30

**Authors:** Ming Zhou, Chenjun Zhai, Kai Shen, Gang Liu, Lei Liu, Jian He, Jun Chen, Yaozeng Xu

**Affiliations:** ^1^Department of Orthopedics, The First Affiliated Hospital of Soochow University, Suzhou 215006, Jiangsu, China; ^2^Department of Orthopedics, Yixing People's Hospital, Yixing 214200, Jiangsu, China; ^3^Department of Orthopedics, The First Affiliated Hospital of Nanjing Medical University, Nanjing 210029, Jiangsu, China

## Abstract

Dysregulation of miRNAs in chondrocytes has been confirmed to participate in osteoarthritis (OA) progression. Previous study has screen out several key miRNAs may play crucial role in OA based on bioinformatic analysis. Herein, we identified the downregulation of miR-1 in OA samples and inflamed chondrocytes. The further experiments revealed that miR-1 played an essential role in maintaining chondrocytes proliferation, migration, antiapoptosis, and anabolism. Connexin 43 (CX43) was further predicted and confirmed to be the target of miR-1, and mediated the promotion effects of miR-1 in regulating chondrocyte functions. Mechanistically, miR-1 maintained the expression of GPX4 and SLC7A11 by targeting CX43, attenuated the accumulation of intracellular ROS, lipid ROS, MDA, and Fe^2+^ in chondrocytes, thereby inhibiting the ferroptosis of chondrocytes. Finally, experimental OA model was constructed by anterior cruciate ligament transection surgery, and Agomir-1 was injected into the joint cavity of mice to assess the protective effect of miR-1 in OA progression. Histological staining, immunofluorescence staining and Osteoarthritis Research Society International score revealed that miR-1 could alleviate the OA progression. Therefore, our study elucidated the mechanism of miR-1 in OA in detail and provided a new insight for the treatment of OA.

## 1. Introduction

Osteoarthritis (OA) is a multi-subtype degenerative joint disease, which is characterized by progressive and irreversible degeneration of articular cartilage and struct4ural changes of subchondral bone [1]. Globally, OA is a highly prevalent and disabling disease that imposes a huge social and economic burden on society [2]. However, there is currently no effective treatment to prevent the occurrence or stop the progression of OA. Further exploration of the pathological mechanism of the occurrence and development of OA, and the search for potential therapeutic targets may reduce the pain of OA patients, improve their quality of life, and relieve medical pressure.

Articular cartilage is an avascular and neural tissue composed primarily of extracellular matrix (ECM, mainly collagen type II and proteoglycans) [3, 4]. Cartilage provides a unique articular surface with a very low coefficient of friction, and the presence of cartilage can effectively conduct mechanical stress from exercise or normal human body load, thereby protecting normal joint tissue [5, 6]. Mature articular chondrocytes have minimal mitotic activity, but maintain a balance between anabolism and catabolism [7]. Chondrocytes are the only cell type in cartilage, therefore, the maintenance of the normal physiological state of cartilage is crucial for the protection of joint function.

Studies have reported that the function of chondrocytes in OA is severely impaired, manifested as cell loss (apoptosis, necrosis, etc.), and the disturbed balance of synthesis and metabolism, resulting in ECM degradation and loss of cartilage layer. These changes lead to the loss of normal joint function and promote the occurrence and progression of OA [8, 9]. MicroRNAs (miRNAs) are noncoding single-stranded RNA about 22 nucleotides (nt) in length, which can inhibit mRNA translation or promote its degradation by interacting with the 3′-untranslated region (3′-UTR) of target genes [10]. Studies have shown that the dysregulation of miRNAs is involved in the occurrence and development of OA by regulating chondrocyte apoptosis and proliferation, ECM metabolism and inflammatory response [11, 12]. Therefore, uncovering the roles of miRNAs and the mechanisms in OA progression is crucial for identifying new OA biomarkers or therapeutic targets.

Chang et al. [13] screened out several potential key miRNAs (miR-1, miR-21, miR-155, and miR-17), which were speculated involve in OA progression, through bioinformatics analysis based on the sequencing of OA samples. Among the differentially expressed miRNAs, whether the deregulation of miR-1 is involved in the progression of OA and its pathogenic mechanism are still unclear. Li et al. [14] found that miR-1 could reduce cardiomyocyte apoptosis by regulating the expression of cardiac apoptosis-related genes in VMC mice. Che et al. [15] preliminarily explored the participation of miR-1 in the progression of OA through human cartilage tissue and Col2a1-Cre-ER^T2^/GFP^fl/fl^-RFP-miR-1 osteoarthritis mouse model, however, the mechanism was lacking. Therefore, this study aims to explore the role and potential mechanism of miR-1 in OA based on the above studies.

Ferroptosis is an iron-dependent cell death distinct from apoptosis, necrosis, autophagy, and other forms of cell death. Ferroptosis is triggered by inactivation of cellular glutathione (GSH)-dependent antioxidant defenses, resulting in accumulation of toxic lipid ROS (L-ROS) [16, 17]. As a new mechanism for regulating cell fate, ferroptosis has been confirmed to be involved in various diseases in recent years, such as tumors [18], cardiovascular diseases [19], and osteoporosis [20]. Miao et al. [21] analyzed the changes of ferroptosis-related indicators in cartilage of OA patients and normal tissue, and found that ferroptosis is closely related to OA. Their study concluded that the downregulation of GPX4 in cartilage of OA patients can increase the sensitivity of chondrocytes to oxidative stress through the MAPK/NF-*κ*B pathway and aggravate ECM degradation. Recently, Sun et al. [22] revealed that the ferroptosis mediated by nuclear receptor coactivator 4 (NCOA4) plays a crucial role in OA progression. However, the regulation of ferroptosis in OA still needs more research to verify.

Moreover, several studies indicated that miRNAs participate in disease progression by regulating ferroptosis. Ding et al. [23] found that miR-182-5p and miR-378a-3p could regulate ferroptosis in ischemia–reperfusion-induced kidney injury. Wang et al. [24] upregulated the expression of miR-149, which reduced the expression of HMGB1, and ameliorated LPS-induced mouse cardiomyocyte injury by inhibiting the ferroptosis pathway. Whether miR-1 can alleviate the progression of OA by regulating chondrocyte ferroptosis requires further studies.

Based on the above speculation, we intend to investigate the following aspects: (1) verify the expression levels of differential miRNAs in chondrocytes of OA patients; (2) explore the effect of miR-1 on the function of OA chondrocytes; (3) investigate the molecular mechanism of miR-1 in regulating chondrocyte function; (4) explore whether miR-1 participate in the regulation of OA through the ferroptosis pathway.

## 2. Materials and Methods

### 2.1. Human Cartilage Tissue and Chondrocyte Isolation

OA articular samples (*n* = 10) were obtained from patients with osteoarthritis who underwent total knee arthroplasty and the normal articular samples (*n* = 4) were collected from patients with trauma who underwent amputation. The harvest of chondrocytes was performed as previously described [25]. In brief, cartilage tissues were obtained from tibial plateau and cut into 1 mm^3^ slices, then the slices were digested in trypsin-EDTA (Gibco, USA) followed by 0.2% collagenase II (Biofroxx) treatment for 10 hr. Mixture was filtrated through 70 *μ*m nylon mesh (Fisherbrand, USA) and centrifuged. Afterward, chondrocytes were cultured in Dulbecco's Modified Eagle's Medium (DMEM) containing 10% fetal bovine serum (Gibco, USA) and 1% penicillin–streptomycin (Sigma–Aldrich, USA). Chondrocytes were detached and split in new culture plates when reaching 70%–80% confluence. This study was approved by the ethics committee of Yixing People's Hospital (Approve no. IRB-2022-RESEARCH-157), and all patients provided informed consent.

### 2.2. RT-qPCR

For the detection of miRNA expression level in cartilage tissues, samples were dissected and ground in liquid nitrogen. Total miRNA was isolated using miRNeasy Mini kit (Qiagen, Germany) following the instructions and the RNA samples were reverse-transcribed into complementary DNA (cDNA) with MiScript Reverse Transcription kit (Qiagen, Germany). qPCR was performed using the TB Green™ Premix Ex Taq™ II kit (Takara, Japan) on ABI 7900 fast real-time PCR system (Applied Biosystems, USA). The expression levels of miRNA were normalized to U6, and all primers were purchased from RiboBio (China).

### 2.3. Western Blot Assay

The proteins of chondrocytes, which were treated differently, were lysed by RIPA buffer containing protease and phosphatase inhibitor cocktail (Beyotime Biotechnology, China). The concentration was measured by BCA kits (Beyotime Biotechnology, China). After being heated with loading buffer, the proteins were separated by SDA-PAGE before transferring to polyvinylidene fluoride (PVDF) membranes (EMD Millipore Corp, USA). Membranes were blocked by 5% BSA followed by primary antibody incubation. After incubation, membranes were washed and treated with secondary antibody for 2 hr at room temperature. The expressions of proteins were visualized by ECL reagent and recorded by Tanon 5200. The antibodies used in this study were anti-collagen II (Proteintech, 28459-1-AP), aggrecan (Proteintech, 13880-1-AP), GAPDH (Proteintech, 60004-1-Ig), SOX9 (Abcam, ab185966), MMP-13 (Abcam, ab51072), cleaved caspase-3 (Abcam, ab32042), BCL-2 (Cell Signaling Technology, 15071), CX43 (Cell Signaling Technology, 3512), GPX4 (Affinity Biosciences, DF6701), and SLC7A11 (Affinity Biosciences, DF12509).

### 2.4. Cell Transfection

Chondrocytes were transfected with 50 nM Agomir-1, Agomir-1 negative control (Agomir NC), Antagomir-1 and Antagomir-1 negative control (Antagomir NC), all purchased from RiboBio, using Lipofectamine 3000 reagent (Invitrogen, USA). CX43 plasmids were constructed by GenePharma (China). The package of virus and the test of titer were performed as described before [26]. 1 × 10^8^ lentivirus-transducing units were used to infect chondrocytes in the presence of polybrene (GenePharma, China).

### 2.5. Cell Proliferation Assays

To detect the proliferation ability of chondrocytes after different treatments, CCK-8 and EdU assays were conducted. Five thousand chondrocytes treated differently were seeded into 96-well plates and incubated at 37°C. At Day 0, 1, 2, and 3, CCK-8 solution (10 *μ*L/well, Dojindo, Japan) were added and cells were further cultured for 4 hr. The absorbance at 450 nm was detected by a microplate reader (Multiskan FC, Thermo Fisher Scientific, USA).

To further assess the proliferation, 1 × 10^4^ chondrocytes treated differently were seeded into 96-well plates. After attachment, chondrocytes were treated with 50 *μ*Mz EdU solution (RiboBio, China) and incubated for another 2 hr. Then, cells were fixed in 4% PFA and stained by Apollo solution according to the manufacturer's instructions. The nucleus was stained with 4′, 6-diamidino-2-phenylindole (DAPI). The images were recorded using fluorescence microscopy, and the rate of positive chondrocytes was measured by ImageJ software.

### 2.6. Cell Migration Assays

Scratch wound healing assay and transwell assay were conducted to measure the migration ability of chondrocytes. In scratch wound healing assay, 2 × 10^5^/well chondrocytes treated differently were seeded into 6-well plates and cultured in complete medium. After 100% confluence, a scratch was made by a 200 *μ*L pipette tip and carefully rinse the cells with PBS thrice. The scratch images were captured by a light microscope (Nikon, Japan) at 0 and 12 hr following scratching. Healing rates of the scratch were analyzed by ImageJ. In transwell assay, 1 × 10^5^ chondrocytes in a volume of 200 *μ*L serum-free medium were seeded in the upper chamber (Corning, USA, pore size: 8 *μ*m), and 600 *μ*L of complete medium was introduced into the lower chamber. After 24 hr incubation, cells in the upper chamber were wiped gently with a cotton swab, and the lower surface was fixed by PFA and stained with 0.5% crystal violet for 1 min. The migrated chondrocytes were observed by microscope and counted using ImageJ.

### 2.7. Cell Apoptosis Assay

To assess the apoptosis of chondrocytes, cells were induced by IL-1*β* and the apoptosis rate was detected using Annexin V-FITC/PI Apoptosis Detection Kit (Vazyme, China) according to manufacturer's instructions. In brief, after transfection, 4 × 10^5^-treated chondrocytes of each group were seeded into 6-well plates. After 70% confluence, the medium was replaced and 10 ng/ml IL-1*β* were added to induce cell apoptosis. Chondrocytes were harvested after 24 hr treatment and resuspended in binding buffer. Then, chondrocytes were stained with 5 *μ*L Annexin V-FITC and 5 *μ*L propidium iodide for 10 min. The apoptosis rate was analyzed using a flow cytometer (CytoFLEX, USA).

### 2.8. Luciferase Reporter Assay

To verify whether CX43 mRNA is the target of miR-1, the luciferase report vectors including pmir-report-CX43 wild-type (WT) and pmir-report-CX43 mutant were transfected into chondrocytes with Agomir-1 or the negative control. Twenty-four hours later, chondrocytes were lysed, and the firefly and renilla luciferase activity were detected by Dual-Luciferase® Assay Kit (Promega, USA).

### 2.9. Intracellular ROS and Lipid ROS Detection

To detect the intracellular ROS and Lipid ROS, fluorescent probes DCFH-DA (Beyotime Biotechnology, China) and C11-BODIPY (MCE, USA) were used following the manufacturer's instructions. In brief, 2 × 10^5^ cells/well chondrocytes were seeded into 6-well plates. After incubation for 24 hr, cells from different groups were washed with PBS and treated with 10 *μ*M DCFH-DA or 5 *μ*M C11-BODIPY for 30 min at 37°C in the dark. Chondrocytes were observed and the images were captured using a fluorescence microscope (Leica, China). In the C11-BODIPY experiment, the red fluorescence associated with the unoxidized dye and the green fluorescence associated with the oxidized dye were respectively excited at 561 and 488 nm.

### 2.10. Detection of Lipid Peroxidation and Fe^2+^ Content

To detect the concentration of MDA (a lipid peroxidation product) in cell lysates, an MDA assay kit (Abcam, USA) was used according to the manufacturer's instructions. An iron assay kit (Abcam, USA) was used to determine the concentration of intracellular ferrous iron level (Fe^2+^) in cell lysates as described before [27]. In brief, samples were washed with cold PBS and homogenized in iron assay buffer. Five microliter iron reducer was added to each standard well and 5 *μ*L of assay buffer was added to each sample, and then the wells were incubated at 37°C for 30 min. After incubation, 100 *µ*L iron probe was added to each well and incubated for 1 hr. The content was measured at 593 nm using a colorimetric microplate reader.

### 2.11. Experimental OA Model

To construct experimental OA model, anterior cruciate ligament transection (ACLT) surgery was conducted as described before [28]. All procedures were approved by the ethical review committee of Soochow University (Approve no. 202206A0038). In brief, male C57BL/6 mice (8 weeks) were anesthetized with pentobarbital (35 mg/kg) and the anterior cruciate ligament of the right joint was transected with a microiris scissor. After operation, mice were treated with buprenorphine (0.05 mg/kg) and gentamicin (5 mg/kg) to prevent pain and infection.

Mice in this study were divided into four groups: Sham group, ACLT group, ACLT + Agomir NC group, and ACLT + Agomir-1 group. In third and fourth group, 5 nmol Agomir NC or Agomir-1 was injected into articular cavity for 3 weeks (twice a week) after surgery. In first and second group, PBS was injected as control. After total of 8-weeks induction, all mice were sacrificed and the joints were collected. Collected joints were fixed in 4% PFA, decalcified in 10% ethylenediaminetetraacetic acid (EDTA) solution, and embedded in paraffin for further experiments.

### 2.12. Histological and Immunofluorescence Staining

The specimens of OA patients and mice were cut into 5 *μ*m, and sections were dewaxed. For histological staining, sections were stained with hematoxylin and eosin (HE), Safranin O/Fast green and Toluidine Blue. The OA progression was evaluated using Osteoarthritis Research Society International (OARSI) score.

For immunofluorescence staining, sections were treated with sodium citrate antigen retrieval solution (Solarbio, China) for antigen retrieval. Sections were then permeabilized with 0.1% Triton X-100 and blocked with 5% BSA. To detect the OA progression, collagen II, aggrecan, and MMP13 primary antibodies were incubated with sections overnight at 4°C. After incubation, sections were washed and treated with fluorescent secondary antibodies. The immunofluorescence was observed by a fluorescence microscopy and the intensity of different groups were analyzed by ImageJ.

### 2.13. Statistical Analysis

GraphPad Prism 8.4.3 was used for statistical analysis. The student's *t*-test was used to compare two groups, whereas one-way analysis of variance (ANOVA) for several groups. All results in this study were presented as the mean ± SD. *p* < 0.05 was considered statistically significant.

## 3. Results

### 3.1. miR-1 Was Downregulated in Osteoarthritis

**Figure 1:**
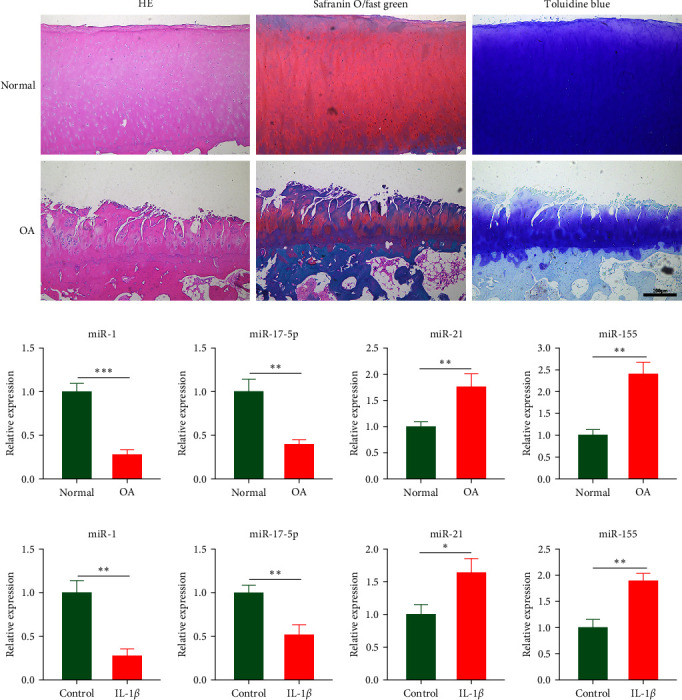
miR-1 was downregulated in osteoarthritis. (a) Histological staining of OA cartilage samples, including HE, safranin O/Fast green and toluidine blue. (b) Verification of the expression levels of potential key miRNAs (miR-1, miR-21, miR-155, and miR-17) in OA samples by qPCR. (c) Detection of the expression levels of potential key miRNAs (miR-1, miR-21, miR-155, and miR-17) in IL-1*β*-stimulated chondrocytes by qPCR. Data were presented as the mean ± SD of at least three replicates. Scale bar = 200 *µ*m.  ^*∗*^*p* < 0.05,  ^*∗∗*^*p* < 0.01 and  ^*∗∗∗*^*p* < 0.001.

Histological staining revealed cartilage erosion and loss of cartilage matrix in OA samples (Figure 1(a)). To verify the expression levels of potential key miRNAs (miR-1, miR-21, miR-155, and miR-17) in OA mentioned by Chang et al. [13] qPCR was conducted. The results indicated that the expressions of miR-1 and miR-17-5p were downregulated, and the expressions of miR-21 and miR-155 were upregulated in OA cartilage (Figure 1(b)). Furthermore, we stimulated chondrocytes with IL-1*β* to obtain in vitro model of inflammation and examined the expression levels of corresponding miRNAs. The results of qPCR showed the same changes of miRNAs as observed in tissue (Figure 1(c)). These results confirmed that several miRNAs predicted in OA cartilage were altered. The role of miR-1 in regulating OA progression was still unclear, however, thus miR-1 was chosen for further study.

### 3.2. miR-1 Played an Essential Role in Regulating Chondrocyte Functions

**Figure 2:**
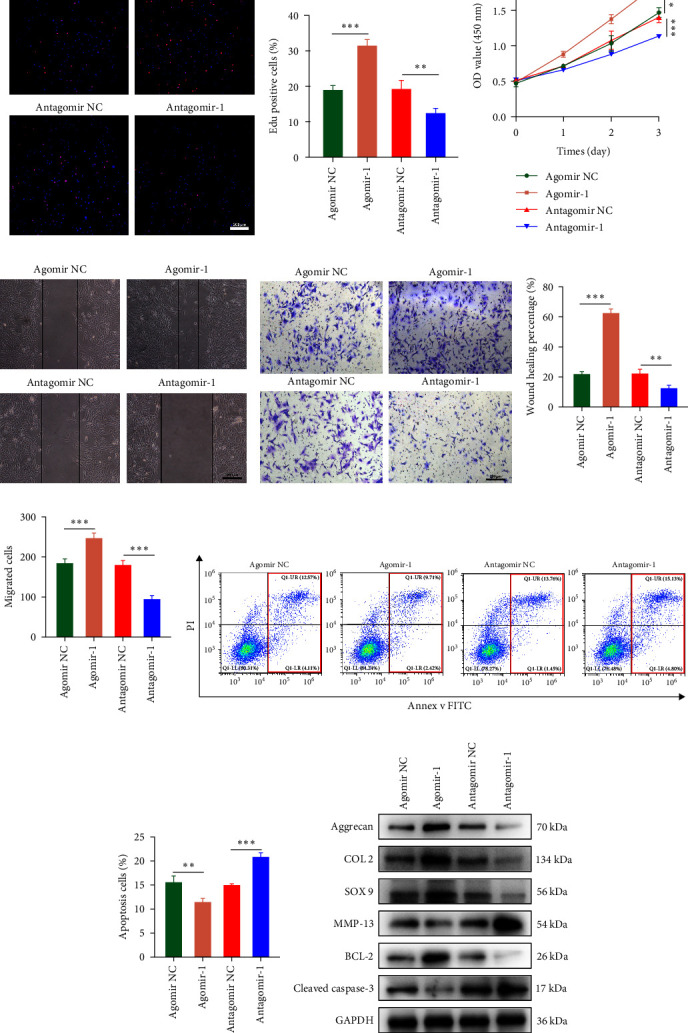
miR-1 played an essential role in regulating chondrocyte functions. (a) Cell proliferation of chondrocytes detected by EdU assay. (b) Quantitative analysis of the EdU assay. (c) CCK-8 assay used to detect the proliferation ability of chondrocytes. (d) Representative images showing the migration ability of chondrocytes by scratch wound healing assay. (e) Representative images showing migrated chondrocytes by transwell assay. (f) Quantitative analysis of scratch wound healing assay. (g) Quantitative analysis of the migrated chondrocytes in transwell assay. (h) Representative images of flow cytometry showing apoptosis rate of chondrocytes using Annexin V/FITC/PI double-staining. (i) Quantitative analysis of the apoptosis rate of chondrocytes. (j) The protein expression levels of aggrecan, Col2, Sox9, MMP13, BCL-2, and cleaved caspase-3 in chondrocytes detected by Western blot assay. Scale bar = 200 *µ*m. Data were presented as the mean ± SD of at least three replicates.  ^*∗∗*^*p* < 0.01 and  ^*∗∗∗*^*p* < 0.001.

To investigate the role of miR-1 in regulating chondrocyte functions, Agomir-1 and Antagomir-1 were used to transfect chondrocytes. As shown in Figure 2(a)–2(b), EdU assay indicated that the upregulation of miR-1 could promote the proliferation of chondrocytes, while the downregulation of miR-1 inhibited the proliferation. Same result was also observed in CCK-8 assay (Figure 2(c)). Furthermore, the migration ability of chondrocytes was tested by scratch wound healing assay and transwell assay. Results indicated that miR-1 could significantly promote the migration of chondrocytes (Figure 2(d)–2(g)). The existence of inflammation could induce chondrocytes apoptosis, thus we also assessed the protection of miR-1 in IL-1*β*-induced chondrocyte apoptosis by flow cytometry. The results showed that the apoptosis rate of chondrocytes was significantly reduced in Agomir-1 group, and increased in Antagomir-1 group (Figure 2(h)–2(i)), indicating miR-1 could protect the chondrocytes from inflammation-induced apoptosis. The Western blot results showed that miR-1 could promote the expression of aggrecan, Col2, SOX9, and BCL-2, while reducing the levels of MMP-13 and cleaved-caspase 3 (Figure 2(j)). These results revealed that miR-1 played an essential in regulating chondrocyte functions.

### 3.3. miR-1 Regulated Chondrocyte Functions by Targeting CX43

**Figure 3:**
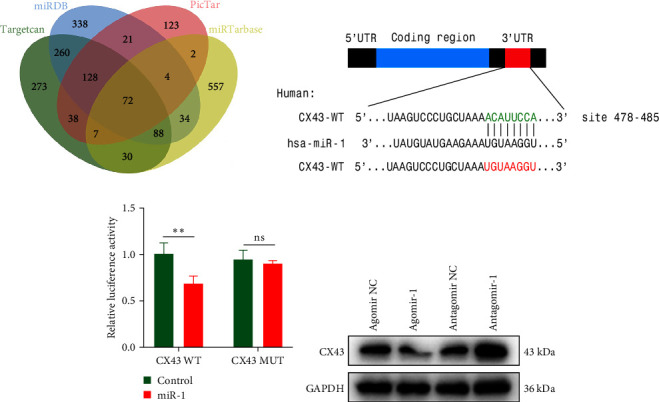
CX43 was the target of miR-1. (a) Venn diagram showing the potential target mRNAs of miR-1, as identified by four different independent microRNA-target-predicting online databases (Target scan, miRDB, PicTar, and miRTarBase). (b) The predicted sequence in the 3′-UTR of CX43 targeted by miR-1. (c) Luciferase reporter assay used to confirm that CX43 is the target of miR-1. (d) Western blot analysis of the expression level of CX43 in chondrocytes transfected with Agomir-1 or Antagomir-1. Data were presented as the mean ± SD of at least three replicates.  ^*∗∗*^*p* < 0.01; ns, no statistical significance.

As we observed above, miR-1 was essential in regulating chondrocyte functions, however, the mechanism was unclear. Four online databases (Target Scan, miRDB, PicTar, and miRTarBase) were used to investigate the target gene of miR-1, and Connexin 43 (CX43) was predicted to be the target (Figure 3(a)). Next, we construct the WT CX43 3′-UTR containing the target sequences, or mutant sequence into luciferase reporter system to verify the prediction (Figure 3(b)). As shown in Figure 3(c), the relative luciferase activity was significantly decreased when cells were co-transfected with miR-1 and CX43 WT, while the suppression was abolished by mutating the target sites in the 3′UTR of CX43. Furthermore, the results of Western blot demonstrated that the expression of CX43 protein was inhibited by miR-1 overexpression, while the miR-1 inhibitor enhanced CX43 expression (Figure 3(d)).

**Figure 4:**
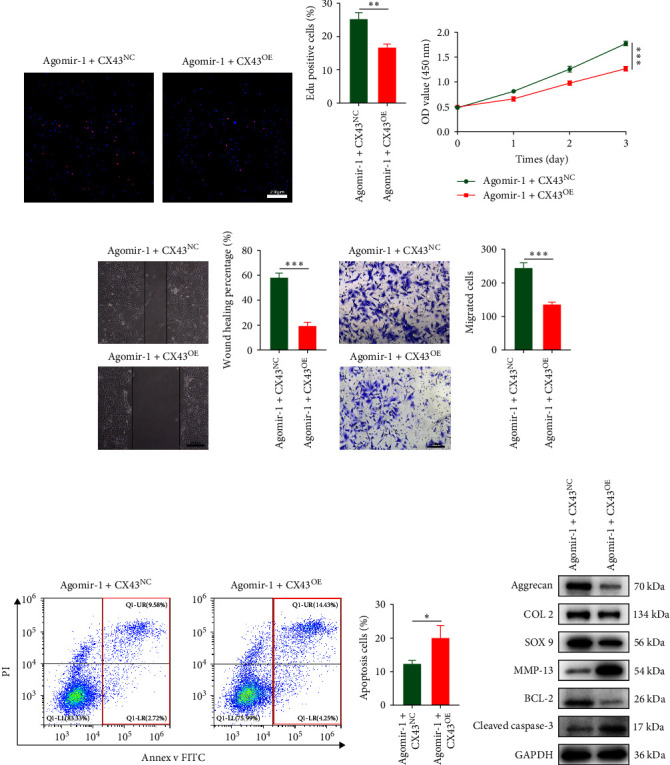
miR-1 regulated chondrocyte functions by targeting CX43. (a–c) EdU and CCK-8 assays used to detect the proliferation ability of chondrocytes. (d–g) Scratch wound healing and transwell assays used to detect the functional role of CX43 on the migration ability of chondrocytes. (h–i) Annexin V/FITC/PI double-staining assay used to verify the role of CX43 in chondrocyte apoptosis. (j) The protein expression levels of aggrecan, Col2, Sox9, MMP13, BCL-2, and cleaved caspase-3 in chondrocytes after CX43 transfection. Scale bar = 200 *µ*m. Data were presented as the mean ± SD of at least three replicates.  ^*∗*^*p* < 0.05,  ^*∗∗*^*p* < 0.01 and  ^*∗∗∗*^*p* < 0.001.

To explore whether miR-1 regulated chondrocyte functions by targeting CX43, chondrocytes were transfected with Agomir-1 and CX43 plasmids (CX43^OE^) or its negative control (CX43^NC^). EdU and CCK-8 assays demonstrated that the overexpression of CX43 significantly attenuated the proliferation ability of chondrocytes (Figure 4(a)–4(c)). As shown in Figure 4(d)–4(g), the percentage of wound healing area and migrated cells were decreased in CX43^OE^ group, indicating the overexpression of CX43 inhibited the migration ability of chondrocytes. Additionally, the apoptosis rate of chondrocytes transfected with CX43 was increased after IL-1*β* stimulation (Figure 4(h)–4(i)). Moreover, Western blot assay revealed that the genes related to chondrogenesis such as aggrecan, Col2, Sox9, and antiapoptotic gene BCL-2 were decreased on CX43^OE^ group, while MMP-13 and cleaved caspase-3 were increased (Figure 4(j)). In summary, above results demonstrated that miR-1 promoted chondrocyte functions by targeting CX43.

### 3.4. miR-1 Inhibited Ferroptosis in Chondrocytes by Targeting CX43, Attenuating Matrix Degradation and Inflammation

**Figure 5:**
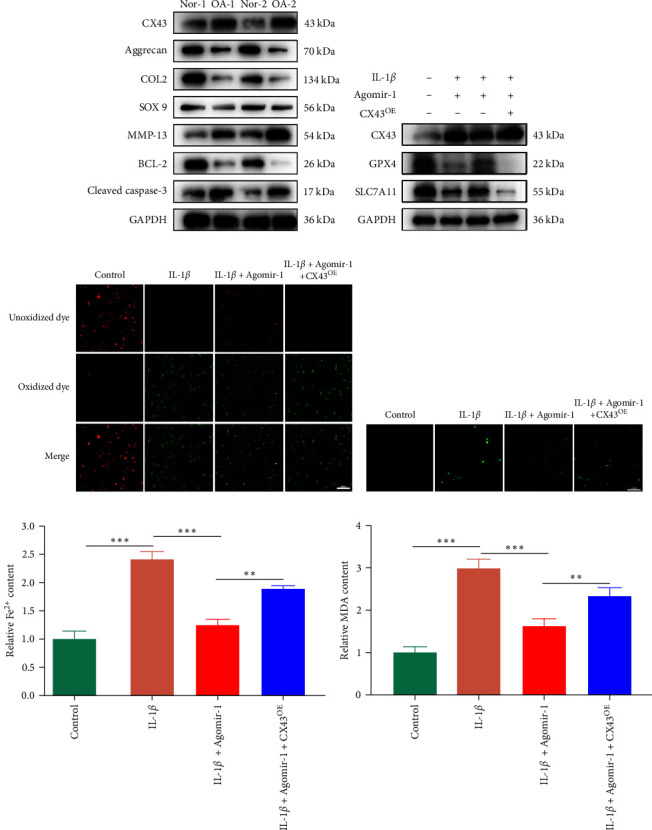
miR-1 inhibited ferroptosis in chondrocytes by targeting CX43, attenuating matrix degradation and inflammation. (a) Western blot assay used to detect the protein expression levels of CX43, aggrecan, Col2, Sox9, MMP13, BCL-2, and cleaved caspase-3 in OA and normal cartilage samples. (b) The expression levels of CX43 and antiferroptosis proteins (GPX4, SLC7A11) in chondrocytes transfected with or without Agomir-1 and CX43. (c) C11 BODIPY fluorescent probe used to detect the level of intracellular lipid-ROS in chondrocytes. Red indicated unoxidized dye, green indicated oxidized dye. (d) Intracellular ROS in chondrocytes detected by DCFHDA. (e–f) Fe^2+^ and MDA concentrations in chondrocytes were measured using the iron assay kit and MDA assay kit. Scale bar = 200 *µ*m. Data were presented as the mean ± SD of at least three replicates.  ^*∗∗*^*p* < 0.01 and  ^*∗∗∗*^*p* < 0.001.

Recently, several studies have demonstrated that ferroptosis plays an important role in the progression of OA [29–31]. Previous study found that the downregulation of CX43 can alleviate cisplatin-induced acute kidney injury by inhibiting ferroptosis [32]. However, whether miR-1 could regulate chondrocyte ferroptosis by targeting CX43 was unclear. First, we detected the expression level of CX43 in OA and normal cartilage samples. Western blot analysis showed that CX43 was elevated in OA samples, accompanied by decreased chondrogenic genes and increased catabolic genes (Figure 5(a)). Next, we transfected chondrocytes with Agomir-1 and CX43 to observe the expressions of ferroptosis-related genes induced by IL-1*β*. As shown in Figure 5(b), CX43 was elevated in chondrocytes accompanied by decreased GPX4 and SLC7A11 after IL-1*β* stimulation compared to untreated group. Agomir-1 treatment could attenuate the upregulation of CX43, however, and rescued the expression of proteins related to antiferroptosis. Interestingly, the ferroptosis of chondrocytes overexpressing miR-1 was aggravated again after CX43 transfection. To further confirm the role of miR-1 and CX43 in chondrocyte apoptosis, intracellular ROS and lipid ROS were detected. As shown in Figure 5(c)–5(d), IL-1*β* treatment could increase the level of intracellular ROS and lipid ROS, while Agomir-1 attenuated the accumulation. However, the overexpression of CX43 blocked the protection of miR-1 in preventing chondrocytes from ferroptosis. In addition, compared with the control group, IL-1*β* stimulated chondrocytes to produce more Fe^2+^ and MDA, and miR-1 reduced the accumulation of Fe^2+^ and MDA in chondrocytes, while CX43 partially abolished the protection of miR-1 (Figures 5(e)–5(f)). In summary, these results demonstrate that miR-1 could modulate chondrocyte ferroptosis by targeting CX43, attenuating the inflammation response and maintaining the homeostasis of chondrocytes.

### 3.5. miR-1 Inhibited OA Progression in Experimental OA Model

**Figure 6:**
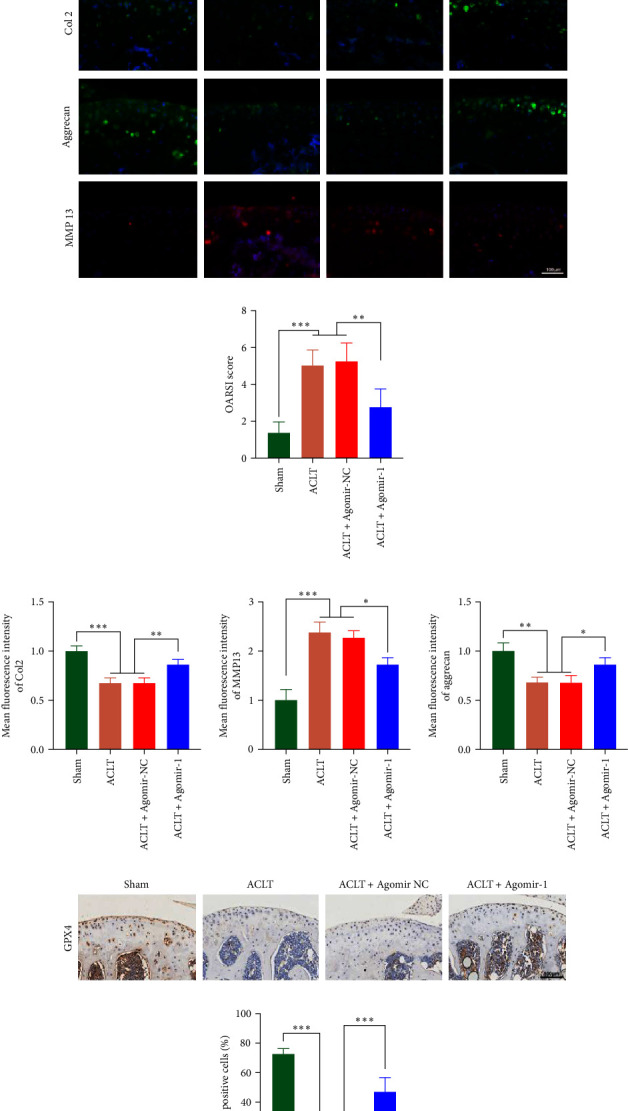
miR-1 inhibited OA progression in experimental OA model. (a) Representative images of safranin O/fast green staining and toluidine blue staining for mice joint after intra-articular injection of Agomir-1. Scale bar = 200 *µ*m. (b) Representative images of immunofluorescence staining of Col2, aggrecan and MMP13 for mice joint after intra-articular injection of Agomir-1. Scale bar = 100 *µ*m. (c) OARSI score for assessing arthritis severity. (d) Quantitative analysis of immunofluorescence staining of Col2, aggrecan and MMP13 for mice joint. (e) Representative images of immunohistochemical staining of GPX4. Scale bar = 100 *µ*m. (f) Analysis of the percentage of GPX4-positive cells. Data were presented as the mean ± SD of at least three replicates.  ^*∗*^*p* < 0.05,  ^*∗∗*^*p* < 0.01 and  ^*∗∗∗*^*p* < 0.001.

To investigate the protective role of miR-1 in OA progression, ACLT surgery was performed to induce OA in vivo, and Agomir-1 was injected. As shown in Figure 6(a), safranin O/fast green and toluidine blue staining revealed that the cartilage in ACLT and ACLT + Agomir NC group appeared collapse, erosion, and ECM loss, while Agomir-1 injection attenuated the damage. Also, the OARSI score of Agomir-1 group was lower compared with ACLT and ACLT + Agomir NC group (Figure 6(c)). In addition, immunofluorescence staining showed that the expressions of Col2 and aggrecan were significantly reduced after surgery, while Agomir-1 injection could partially reverse the degradation of matrix (Figures 6(b) and 6(d)). In contrast, miR-1 simultaneously decreased the expression of MMP13. These findings confirmed that miR-1 could inhibit the degradation of OA cartilage and attenuate OA progression. Furthermore, the results of immunohistochemical staining showed that the expression of GPX4 in the Agomir-1 group was significantly higher than that in the ACLT and ACLT + Agomir NC groups, indicating that Agomir-1 could alleviate the ferroptosis of chondrocytes (Figures 6(e) and 6(f)).

## 4. Discussion

As a common degenerative joint disease, the incidence of OA is increasing in recent years [33]. The occurrence of OA not only threatens human health, but also increases the economic burden of society. OA is a disease that involves dysfunction of various tissues, including cartilage, subchondral bone, and synovium [34]. Among them, the research on the mechanism of cartilage lesions is particularly extensive. However, at this stage, the therapeutic targets for alleviating the progression of OA need to be further explored.

Chondrocytes are the only cells in cartilage, and the changes in their functions may be an important factor leading to cartilage damage. Recently, studies have found that many factors mediate the genetic changes of chondrocytes, including the stimulation of inflammatory factors [35], the load of mechanical stress [36], and age-related changes [37], etc. Among the genetic changes, dysregulation of miRNAs has been implicated in the progression of OA [38]. Cao et al. [39] found miR-214-3p was downregulated in inflamed chondrocytes and OA cartilage and demonstrated that the decreased miR-214-3p can activate the NF-*κ*B signaling pathway and aggravate the development of OA through targeting IKK*β*. Also, Zhang et al. [40] revealed that the expression of miR-17 is downregulated in OA chondrocytes and the deficiency of miR-17 contributes to OA progression. These studies demonstrate that miRNAs are essential in regulating the development of OA. Recently, Chang et al. [13] screened out several potential key miRNAs may involve in OA progression based on bioinformatics analysis, including miR-1, miR-21, miR-155, and miR-17. Thus, we verified the changes of predicted miRNAs in OA cartilage and inflamed chondrocytes. Our results confirmed that the predicted miRNAs were indeed changed, and the participation of miR-1 involved in OA was still unclear, so we chose miR-1 for further study.

In the present study, we demonstrated that the expression of miR-1 was downregulated in OA cartilage and inflamed chondrocytes. miR-1 was confirmed to involve in myocyte proliferation and differentiation before [41]. Herein, we found that miR-1 could promote the functions of chondrocytes, including proliferation, migration, antiapoptosis, and anabolism. miRNAs regulate gene expression posttranscriptionally and regulate cellular biological processes [42]. miRNAs can exert their functions by combining the 3′-UTR of target mRNA and regulating the expressions of genes [43]. In our study, we identified CX43 as the target gene of miR-1 by using miRNA-target predicting online databases and verified the prediction by luciferase reporter assay. Further studies demonstrate that the functional promotion of miR-1 in chondrocytes was reversed by CX43 overexpression, indicating miR-1-mediated repression of CX43 is critical for maintaining chondrocyte functions.

Several forms of chondrocyte death can be induced by inflammation, oxidative stress, including apoptosis [44], autophagy [45], and necroptosis [46]. As a new form of cell death participated in the pathology of OA, ferroptosis has been identified to have an important role in OA [47]. Inspired by Yu et al. [32], we sought to explore whether miR-1 attenuated the ferroptosis of chondrocytes by targeting CX43. Our data revealed that miR-1 can maintain the expression of antiferroptosis genes and reduced the accumulation of ROS, lipid ROS, Fe^2+^, and MDA in IL-1*β* stimulated chondrocytes. CX43 overexpression abolished the protection of miR-1, however, aggravated the ferroptosis of chondrocytes. These results demonstrated that miR-1 can regulate chondrocyte ferroptosis by targeting CX43.

To further confirm the protective effect of miR-1 on OA progression in vivo, experimental OA mode was conducted and Agomir-1 was injected into articular cavity. Histological and immunofluorescence staining revealed that the injection of Agomir-1 could significantly protect the cartilage from matrix degradation and mitigate cartilage erosion, which also confirmed by OARSI score. However, the in vivo experiments in this study are still lacking, the protective effect of miR-1 on the ferroptosis activator Erastin induced arthritis needs further verification.

In summary, the findings of this study demonstrated that miR-1 could inhibit the ferroptosis of chondrocytes and attenuate OA progression by targeting CX43.

## 5. Conclusion

In conclusion, we found the dysregulation of miR-1 in OA samples and inflamed chondrocytes, and demonstrated that miR-1 can promote the proliferation, migration, antiapoptosis, and anabolism of chondrocytes by targeting CX43. Further experiments confirmed that miR-1/CX43 regulated the functions of chondrocyte by inhibiting ferroptosis and alleviates osteoarthritis progression.

## Data Availability

The data presented in this study are available on request from the corresponding author.
